# Experimental Investigation of Effect of L-Profile Hybrid Aluminium/GFRP to the Axial and Lateral Characteristic

**DOI:** 10.3390/polym15051137

**Published:** 2023-02-24

**Authors:** Ariyana Dwiputra Nugraha, Daffa Alandro, Arif Kusumawanto, Endro Junianto, Budi Perwara, Vishnu Vijay Kumar, Gil Nonato C. Santos, Jayan Sentanuhady, Rachmadi Norcahyo, Muhammad Akhsin Muflikhun

**Affiliations:** 1 PLN Research Institute, Jl. Duren Tiga Raya No.102, RT.8/RW.1, Duren Tiga, Kec. Pancoran, Kota Jakarta Selatan, Daerah Khusus Ibukota Jakarta 12760, Indonesia; 2 Mechanical and Industrial Engineering Department, Gadjah Mada University, Jalan Grafika No. 2, Yogyakarta 55281, Indonesia; 3 Department of Architecture and Planning, Gadjah Mada University, Jalan Grafika No. 2, Yogyakarta 55281, Indonesia; 4 Research Center for Electrical Power and Mechatronics—National Research and Innovation Agency (BRIN), Jl. Sangkuriang, Dago, Kecamatan Coblong, Kota Bandung, Jawa Barat 40135, Indonesia; 5 College of Design and Engineering, National University of Singapore, 9 Engineering Drive 1, #07-26 EA, Singapore 117575, Singapore; 6 Department of Ocean Engineering, Indian Institute of Technology Madras, Chennai 600036, India; 7 Physics Department, De La Salle University, 2401 Taft Ave, Malate, Manila 1004, Philippines

**Keywords:** hybrid composite, crashworthiness, aluminium, GFRP, compression test

## Abstract

The current study investigates the effect of a hybrid L-profile aluminium/glass-fiber-reinforced polymer stacking sequence under axial and lateral compression loads. Four stacking sequences are studied: aluminium (A)—glass-fiber (GF)—AGF, GFA, GFAGF, and AGFA. In the axial compression test, the aluminium/GFRP hybrid tends to crush in a more progressive and stable failure than the net aluminium and net GFRP specimens, with a relatively more stable load-carrying capacity throughout the experimental tests. The AGF stacking sequence was second, with an energy absorption of 145.31 kJ, following AGFA at 157.19 kJ. The load-carrying capacity of AGFA was the highest, with an average peak crushing force of 24.59 kN. The second-highest peak crushing force, 14.94 kN, was achieved by GFAGF. The highest amount of energy absorption, 157.19 J, was achieved by the AGFA specimen. The lateral compression test showed a significant increase in load-carrying and energy absorption capacity in the aluminium/GFRP hybrid specimens compared to the net GFRP specimens. AGF had the highest energy absorption with 10.41 J, followed by AGFA with 9.49 J. AGF also had the highest peak crushing force with 2.98 kN, followed by AGFA with 2.16 kN. The most crashworthy stacking sequence among the four variations tested in this experimental research was the AGF stacking sequence because of its great load-carrying capacity, energy absorption, and specific energy absorption in axial and lateral loading. The study provides greater insight into the failure of hybrid composite laminates under lateral and axial compression.

## 1. Introduction

Safety is always the main priority in the design of any vehicle. Crashworthiness is one of the indicators of vehicle safety, especially in the event of crashing to protect the occupants from severe injury. Conventional thin-walled metallic structures have been studied intensively to enhance crashworthy performance over the past decades [1,2,3,4]. Concerns about environmental sustainability have led to a tightening of regulations on vehicle emissions and mining activities. These factors have influenced the ever-growing demand for developing novel, more environmentally friendly structures and materials. For this reason, fiber-reinforced composite materials such as GFRP and CFRP have gained emergent interest in development as crash energy-absorbing components for a vehicle. Fiber-reinforced composites are known for their light weight and remarkable mechanical properties. Various research has proven that, from a crashworthiness perspective, the load-carrying and energy absorption capacities of thin-walled composite structures are superior to those of conventional metals. However, every material has its shortcomings. Fiber-reinforced composites have some noticeable shortcomings despite their superior mechanical properties and lightweight features. Relatively, they have higher costs, and their material brittleness and catastrophic failure modes are the major concerns that limit these materials’ implementation as energy-absorbing components in the automobile industry. Therefore, fiber-reinforced composite structures have not been recommended to be used alone. Instead, it is more common for them to be used in a metal/composite hybrid form in which metal structures are combined with the composite to obtain the beneficial features of both materials [5,6]. Metals and composites have different mechanisms for absorbing energy. The metal component provides crushing stability, ductility, and plasticity. For instance, aluminium is a light material with a high modulus elasticity that can absorb more force before the buckling phenomenon. Meanwhile, a fiber composite component provides high strength and a high stiffness-to-weight ratio. It is often used as a reinforcement structure for various components. Composites tend to have low elasticity, which can cause low energy absorption [5,6,7]. The energy absorption of composite materials mainly occurs by various forms of failure resulting from delamination, fracture, fiber debonding, matrix cracking, etc. The failure mode of hybrid materials is an area of increasing importance [7,8,9,10,11,12,13].

The current study employs experimental works combining aluminium and GFRP. Aluminium is prone to fracture due to its low strength and elongation. Meanwhile, GFRP is a brittle material, which results in low plasticity. Thus, combining these two materials, and therefore the mechanical properties of each material, will display their advantages and offer higher energy absorption capability. The materials have their uniqueness (high stiffness in GFRP and the flexure ability of aluminium), but the hybrid laminate combines their properties [14,15]. Stepwise loading studies of GFRP have been reported previously, where matrix debonding, layer delamination, and fiber breakage were found to be the main factors of failure [10]. Load displacement due to the tensile loading of composites has also been experimented with, and can explain the composite’s behavior after the test [16].

GFRP is so sensitive to compression that it is prone to buckle under compressive loading. The energy absorption ability of sandwich panels in GFRP composites tends to be low [17]. On the other hand, metals have been used as additional reinforcement to prevent the failure of GFRP sandwich panels during compression loading. Improvements in crashworthiness can be made simply by re-arranging the material structure, which can provide a significantly higher level of crashworthiness [18]. The specimen’s profile also significantly impacts durability and strength for loading. A different profile will provide a different result in the test. A multilayer sandwich composite material has better energy absorption than a composite layer material [19,20,21]. Combining these theories allows the best result to be obtained by optimizing the specimen profile with multilayer materials.

The literature shows that metal/composite hybrid materials are better, and validated data has been obtained to support this research. Sun et al. [22] compared the crashworthiness of empty circular CFRP tubes with CFRP/aluminium/steel hybrid tubes filled with aluminium foam and aluminium honeycomb under an axial quasi-static compression test. The result was that the hybrid tubes underwent a more progressive and stable crushing, with noticeable advantages in crashworthiness. Obradovic et al. [23] developed analytical, numerical, and experimental research on the energy-absorbing composite structures where tests were done on simple tubular structures initially and then on the more complex geometry of the nose cone Formula SAE racing car. Good agreement was achieved between the simple tubular structure and the nose cone, supporting that the experimental test on more simple geometry effectively represents the crashworthiness of the material. Qiang et al. [24] conducted a quasi-static axial compression test to investigate the energy absorption of the bio-inspired multi-cell CFRP and square aluminium tubes. The test result shows that, with the same cells, the peak force, mean crushing force, and specific energy absorption (SEA) of the CFRP tubes were higher than those of the Al tubes, making CFRP tubes a more favorable choice for energy-absorbing vehicle structures.

S. Li et al. [25] conducted the experimental test and numerical simulation to investigate the crashworthiness characteristics of four different aluminium/CFRP or aluminum/GFRP hybrid tubes. Two types of stacking sequences of each material were tested. The load-carrying capacity of the aluminum/composite hybrid tubes with an inner composite tube was substantially higher than that of the hybrid tubes with an outer composite counterpart. The effect of stacking sequences was further supported by this research, where the crashworthiness of aluminium/GFRP with an inner composite tube is higher than that of aluminium/CFRP with an outer composite tube despite having inferior mechanical properties in general. Gowid et al. [26] studied the effect of adding woven fiber laminates and fiber steel sandwich laminates on PVC polymer tubes. Four normal and hybrid reinforced configurations were evaluated and compared. Crashworthiness characteristics, including strength and energy absorption capacity, were identified using quasi-static axial compression tests. The results showed that the tube reinforced with a 1 mm steel layer sandwiched between two layers and four layers of woven glass-fiber had the highest SE and CFE, while the tube reinforced with only seven layers of glass-fiber has the highest Initial Peak Load (IPF). This research shows the varied behaviors of metal/composite hybrid tubes. Sebaey T et al. [27] investigated the influence of thermal aging on the compression properties of CFRP composite rectangular tubes under a quasi-static lateral compression test, where polyurethane was the reinforcement in the rectangular tubes. The aging process was carried out at 70 °C for one, seven, and 14 days and at 100 °C for one, seven, and 14 days. The result showed a gradual increase in each of the specimens. W. Zuo et al. [28] analyzed the crashworthiness and failure process of CFRP tubes exposed to hydrothermal aging and high temperatures. The crashworthiness features rapidly declined as temperature and moisture absorption rates rose. The temperature and crashworthiness indexes showed some nonlinear relationships.

The current study investigates the stacking sequence of an L-shaped profile hybrid composite of aluminium and fiber-glass. Varying the stacking sequence can give different results regarding failure behavior. Adding metal reinforcement to the composite is expected to add more strength to the specimen and can increase its crashworthiness. The main objective of this study is to determine the most effective stacking sequence in terms of crashworthiness to promote more extensive application of it in the automotive industry. Common crashworthiness indicators such as peak crushing force (PCF), average crushing force (F_avg_), crushing force efficiency (CFE), energy absorption (EA), and specific energy absorption (SEA) were calculated to indicate the crash performance. Axial and lateral quasi-static compression tests were performed on four types of aluminium/GFRP hybrid composite stacking sequences. During the compression test, specimen deformation was captured every 5 mm of displacement and discussed to study the crushing behavior of each stacking sequence.

## 2. Materials and Methods

### 2.1. Materials and Specimen Preparations

**Figure 1:**
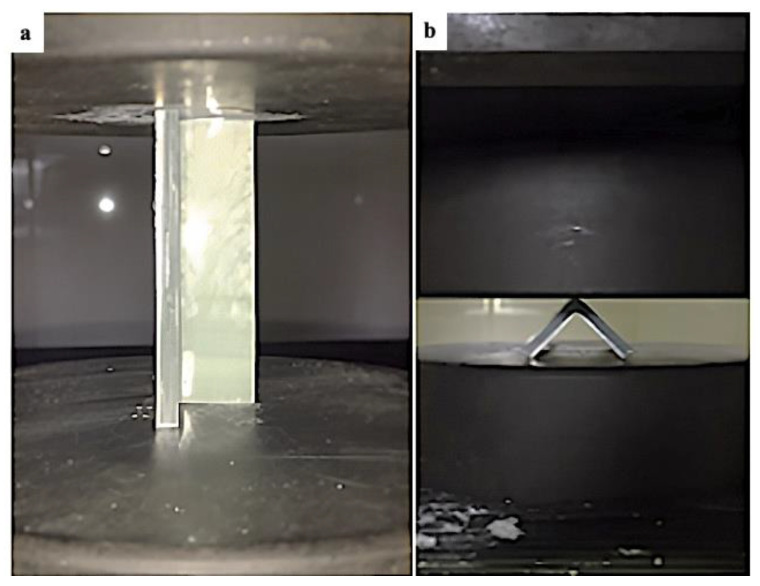
(**a**) Axial and (**b**) lateral specimen position.

**Figure 2:**
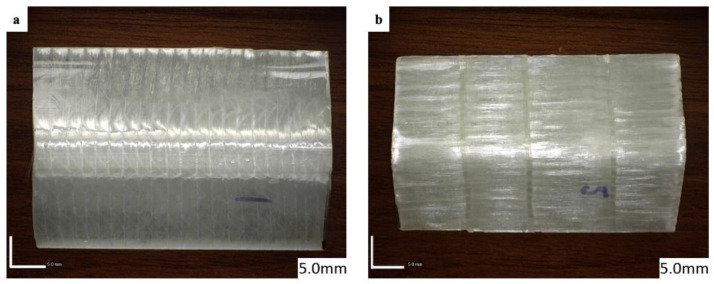
(**a**) Axial and (**b**) lateral fiber orientation.

The axial and lateral compression test was performed in this study, where net aluminium, net GFRP, and four variations of aluminium/GFRP hybrid specimens of each type of test were fabricated and tested to investigate their crushing characteristics. The difference between the two types of compression tests is how the test specimen is positioned on the flat plate of the universal testing machine (UTM) and the orientation of the glass-fiber. Figure 1 shows the different positions of the specimens in the axial and lateral compression test, respectively, and Figure 2 shows the difference in fiber orientation.

**Figure 3:**
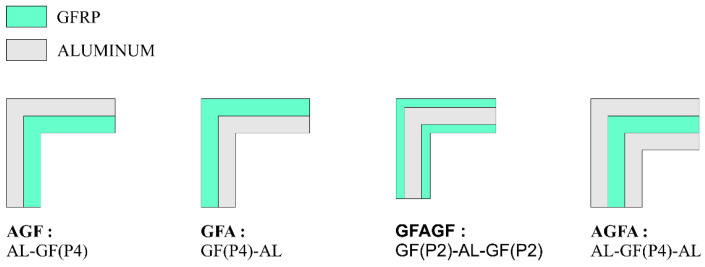
Schematic representation of the stacking sequence.

Each variation of the stacking sequence consisted of four layers of unidirectional continuous glass-fiber sheets as its reinforcement component. As a metal/composite hybrid, the matrixes of the specimens were a combination of polymer and metal. For the polymer component, Bisphenol-A—Epichlorohydrin epoxy resin was activated with Cycloaliphatic Amine epoxy hardener. The resulting matrix would hold the fibers and form a glass-fiber-reinforced-polymer (GFRP) composite. For the metal matrix component, L-profile Aluminium Alloy 3103 with a thickness of 0.8 mm was used. Figure 3 shows an illustration of each variation’s stacking sequence.

**Figure 4:**
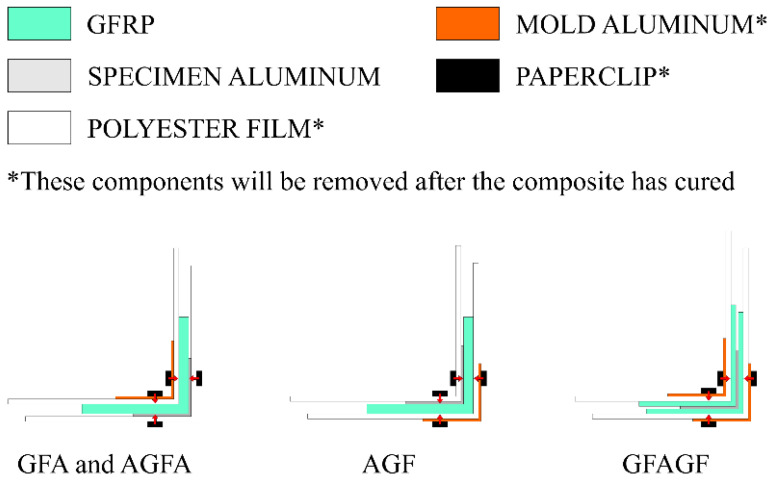
Schematic representation of the specimen curing setup.

The hand lay-up method was employed to produce the specimens for the compression test. The epoxy resin was mixed with the hardener inside a disposable plastic cup with a mixing ratio of 2:1. Then, the resin mixture was stirred thoroughly and placed inside a 74 kPa vacuum desiccator for 10 min for degassing. A flat surface was covered with a sheet of polyester film for the easy removal of the composite post curing. After every layer was greased evenly with resin, the aluminium bar was placed above the resin-covered glass-fiber sheets. The glass-fiber sheets were bent so that they covered every part of the aluminium’s surface. The polyester film was placed on both sides of the hybrid composite for better resin impregnation during curing. Another aluminium bar was placed on the GFRP side to act as a mold. Paper clips were used to hold every component together during the curing process and to apply even pressure on the composite. Figure 4 provides a better understanding of the curing setup.

**Figure 5:**
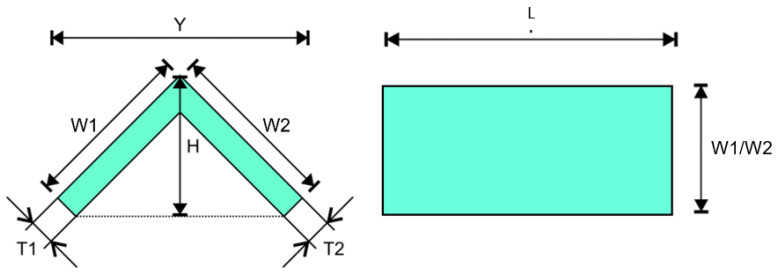
Specimen measurements.

**Figure 6:**
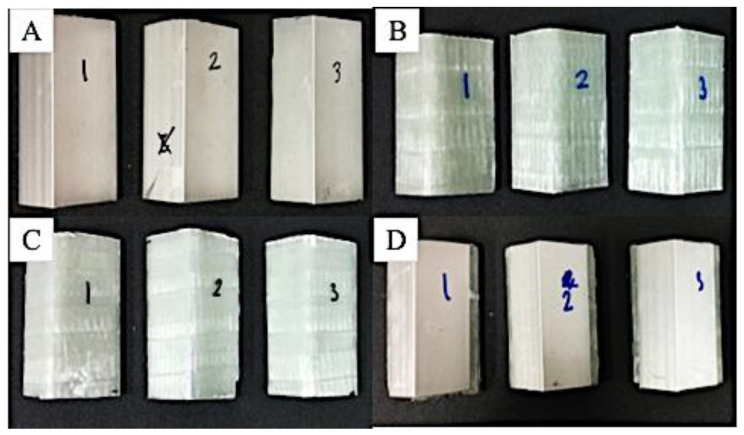
Axial compression test specimens. (**A**) Aluminium L-Profile; (**B**) GFRP Profile; (**C**) Hybrid laminates with GFRP attached on the outer surface of L-Profile; (**D**) Hybrid laminates with GFRP attached on the inner surface of L-Profile.

**Figure 7:**
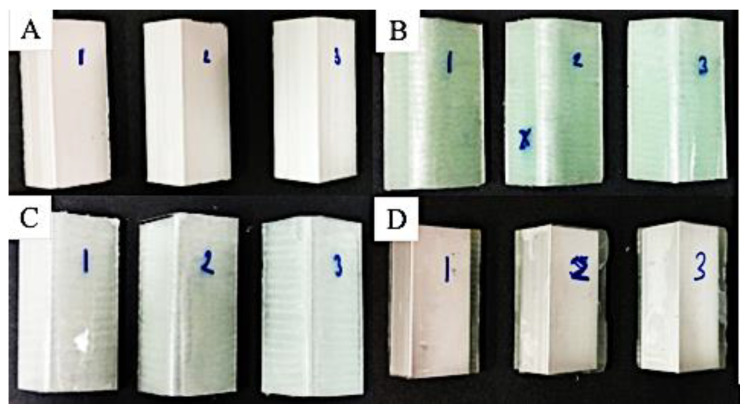
Lateral compression test specimens. (**A**) Aluminium L-Profile; (**B**) GFRP Profile; (**C**) Hybrid laminates with GFRP attached on the outer surface of L-Profile; (**D**) Hybrid laminates with GFRP attached on the inner surface of L-Profile.

The composite was left to cure at room temperature for at least 24 h. Paper clips, polyester film, and the mold aluminium were removed after curing. The hybrid composites were cut into specimens in accordance with the testing dimension using a coolant-aided cut-off saw, and the excess fibers were trimmed away. As shown in Figure 5, Table 1 and Table 2, the specimen dimensions and weight were measured prior to testing. The theoretical volume fraction of each specimen was also calculated with the following equation:

(1) $$V_{f}=\frac{\rho_{f}.w_{f}}{\left(\rho_{m}.W_{m}+\rho_{f}.W_{f}\right)}$$

where *V_f_* denotes the volume fraction of fibers, *W_f_* the weight of fibers, *W_m_* the weight of the matrix, *ρ_f_* the density of fibers, *ρ_m_* the density of the matrix. Three samples with the best quality of each variation were selected out of the five samples and were shown in Figure 6 and Figure 7.

### 2.2. Compression Test and Calculations

A quasi-static compression test was performed to investigate the crashworthiness indicators of the aluminium/composite hybrid specimens. A Gotech GT-7001-LC50 universal testing machine (UTM) was used to conduct the compression test. The hybrid samples were placed on the fixed lower platen, and the moving upper platen was set on a constant crosshead speed of 4 mm/min. the pre-set final displacement of the crosshead was set at 20 mm for the axial compression test and 10 mm for the lateral compression test. During the compression test, a photo of the specimen was taken every 5 mm of crosshead displacement to document the crushing behavior of each sample. Common crashworthiness indicators such as peak crushing force (PCF), average crushing force (F_avg_), crushing force efficiency (CFE), energy absorption (EA), and specific energy absorption (SEA) were calculated to indicate the crash performance of each specimen in the respective type of compression testing. Peak crushing force and average crushing force could be obtained directly from the experimental force–displacement results with the help of Microsoft Excel’s features, while the other crashworthiness metrics were calculated as follows:

(2) $$EA=\overset{d}{\underset{0}{\int }}F\left(x\right)dx$$

(3) $$SEA=\frac{EA}{M}\;$$

(4) $$CFE=\frac{Favg}{PCF}$$

where *d* denotes the displacement and *M* represents the mass of the specimen measured earlier.

## 3. Results and Discussion

The results from the compression tests (axial and lateral) of all specimens are listed in the below section for each type of specimens. There are at least three samples in each combination with similar properties were used in testing. The value of each sample in one type is given in the Appendix A.

### 3.1. Experimental Result of Axial Compression Test

**Figure 8:**
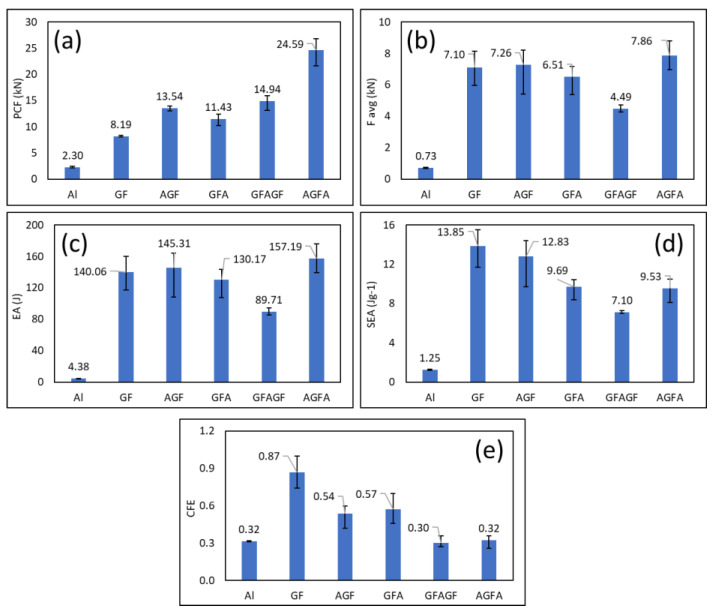
Axial crashworthiness indicators of each specimen load and displacement curve of (**a**) peak crushing force (PCF), (**b**) F average, (**c**) energy absorption (EA), (**d**) specific energy absorption (SEA), (**e**) crush force efficiency (CFE).

**Figure 9:**
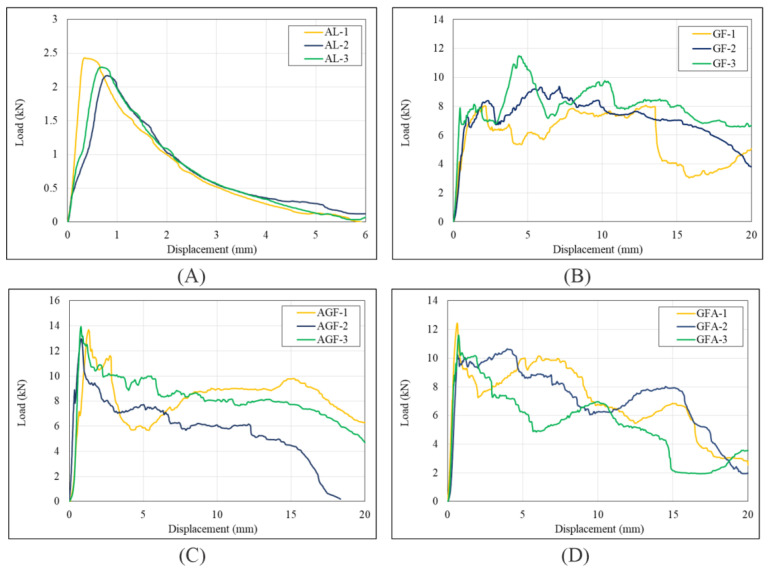

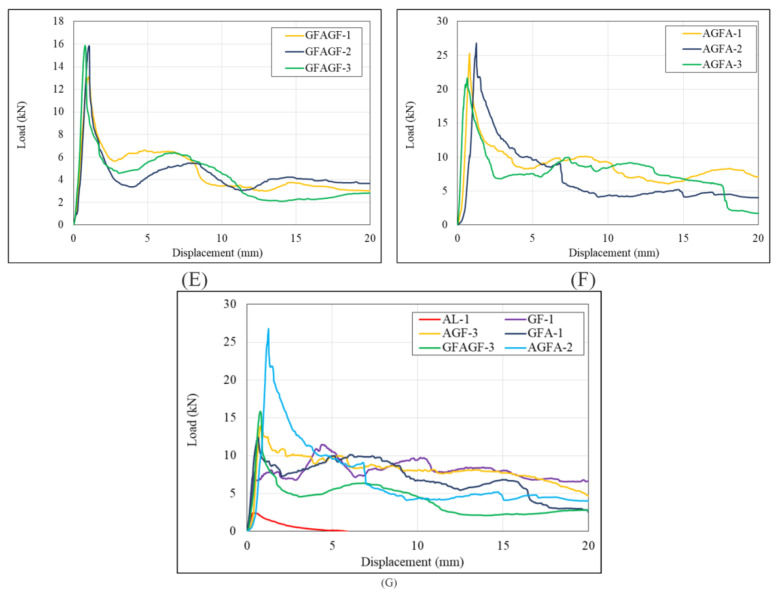
Load and displacement curve of (**A**) net aluminium_,_ (**B**) net GFRP (**C**) AGF, (**D**) GFA, (**E**) GFAGF, (**F**) AGFA, (**G**) Combined.

The load-displacement results from the axial compression test are presented in Figure 8 and Figure 9. The peak crushing force (Figure 8a) showed that the highest value was 24.59 kN (from AGFA) and the lowest was 2.3 kN (from Al). Figure 8b shows the peak average at 7.86 kN and the lowest at 0.73 kN. The highest values of energy absorption and specific energy absorption were 157.19 J and 13.85 J, respectively, as shown in Figure 8c,d. Crush force efficiency in axial compression was observed, with 0.87 as the highest peak. The detailed load-displacement data of all combinations are described in Figure 9.

The axial compression load-displacement curves of the net aluminium, the net GFRP, and four different variations of the hybrid composite are plotted in Figure 8. It was observed that each variation of the hybrid had a reasonably similar curve pattern where the force increased linearly until it reached the peak crushing force as the critical turning point before the fluctuation started to go downwards towards the end of the test. Each specimen also came to the peak crushing force shortly after the test was initiated, where the displacement was not greater than 1.5 mm. The net GFRP and the net aluminium specimens demonstrated a unique compression behavior. The aluminium specimens showed an inverted V-shape in the load and displacement curve where force rose to the peak and went back to 0 with minimal fluctuation. Meanwhile, the net GFRP specimens showed the opposite behavior, where huge fluctuation could be observed throughout the test. The peak force of the GFRP specimens was also somewhat unclear, as the crushing behavior of GFRP was quite catastrophic. From the axial testing results, the net GFRP specimens showed a remarkable energy-absorbing capacity compared to the hybrid specimens. Still, they lacked load-carrying ability, as seen from their lower PCF value. The most noticeable effect of adding the thin aluminium structure on the L-profile GFRP was the increase in the peak crushing force, as it could be seen that every hybrid specimen had a higher PCF than the net GFRP specimens. Due to the brittleness of GFRP, the net GFRP specimens had catastrophic crushing behavior. The addition of aluminium accommodated this problem. A significant difference in load-carrying and energy-absorption capacity could be observed between the aluminium specimens and the GFRP hybrid.

Comparing the hybrid specimens, the AGF and GFA specimens were better at retaining the peak force, as seen from their curves where, after the peak crushing point was reached, the force decreased more gradually than in the other two variations. On the contrary, specimen AGFA and GFAGF dropped significantly after reaching the peak crushing force.

The load-carrying capacity of AGFA was considerably higher than that of the other three variations, with an average peak crushing force of 24.59 kN, because only AGFA has two bars of aluminium supporting its structure instead of one. The GFAGF specimens achieved the second-highest peak crushing force, averaging 14.94 kN. In terms of energy absorption capacity, the AGF specimen’s average energy absorption was very close to that of AGFA, and AGF-1 and AGF-3 had higher energy absorption than the average AGFA, despite having only a single-piece aluminium structure. However, when comparing the specific energy absorption capacity where weight was considered, both AGF and GFA yielded a higher value on average than AGFA because of the extra weight of the other aluminium. This could also be seen from the force-displacement curves, as mentioned before, where AGF and GFA were better at retaining peak force, resulting in better energy absorption and higher crush force efficiency (CFE). This indicates that the AGF sequence is more favorable for vehicle crash energy-absorbing components, and GFAGF is the least promising.

**Figure 10:**
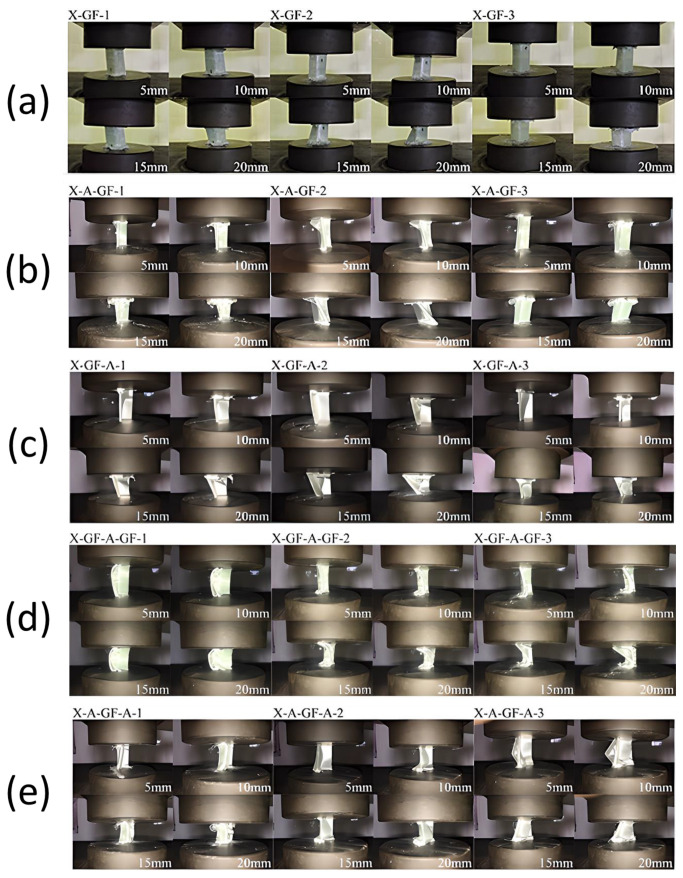
Axial deformation history of (**a**) net GFRP, (**b**) AGF, (**c**) GFA, (**d**) GFAGF, (**e**) AGFA.

Figure 10 presents the axial crushing processes of the net GFRP and the aluminium/GFRP hybrid specimens. Three samples of each variation were tested, and photos were taken every 5 mm crushing distance (5 mm, 10 mm, 15 mm, 20 mm). During the axial compression test, similar failure modes could be observed. On the hybrid specimens, delamination between the aluminium and GFRP started from one of the corners. As the displacement increased, the delamination propagated further, separating the aluminium and GFRP. Delamination within fibers of the GFRP also occurred, where the fibers started to bloom and shatter. The AGF and AGFA specimens tended to retain their standing position during the crushing because of the outer aluminium holding the GFRP in place. This crushing behavior provided better strength and energy absorption than GFA, where the GFRP tended to fall off to the outer side of the L because of the absence of external aluminium. In the GFAGF specimens, delamination was initiated earlier and propagated wider along the body of the specimens. More elastic bending could also be seen in GFRP because of its having an individually thinner GFRP.

### 3.2. Experimental Result of Lateral Compression Test

**Figure 11:**
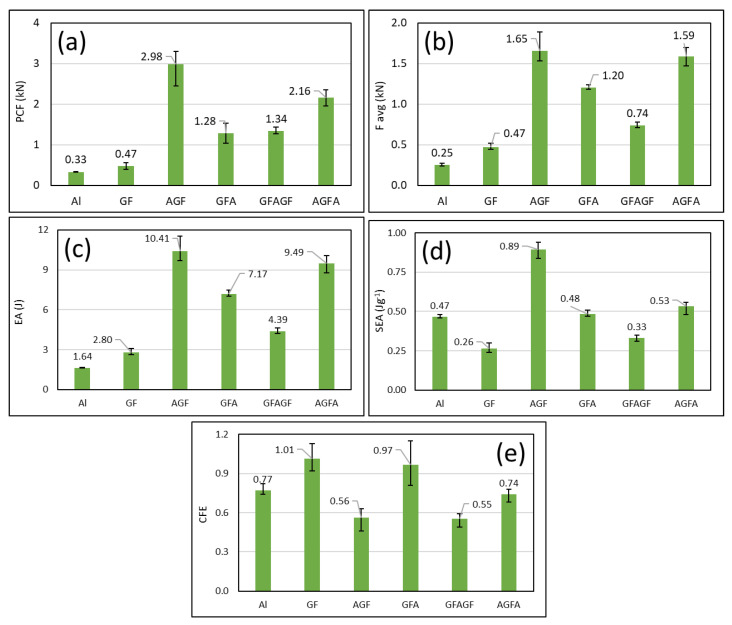
Lateral crashworthiness indicators of each specimen load and displacement curve of (**a**) peak crushing force (PCF), (**b**) F average, (**c**) energy adsorption (EA), (**d**) specific energy absorption (SEA), (**e**) crush force efficiency (CFE).

**Figure 12:**
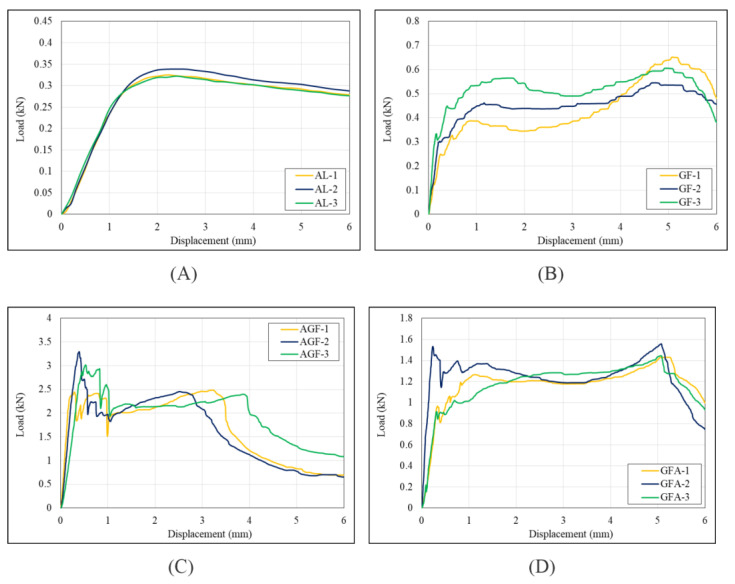

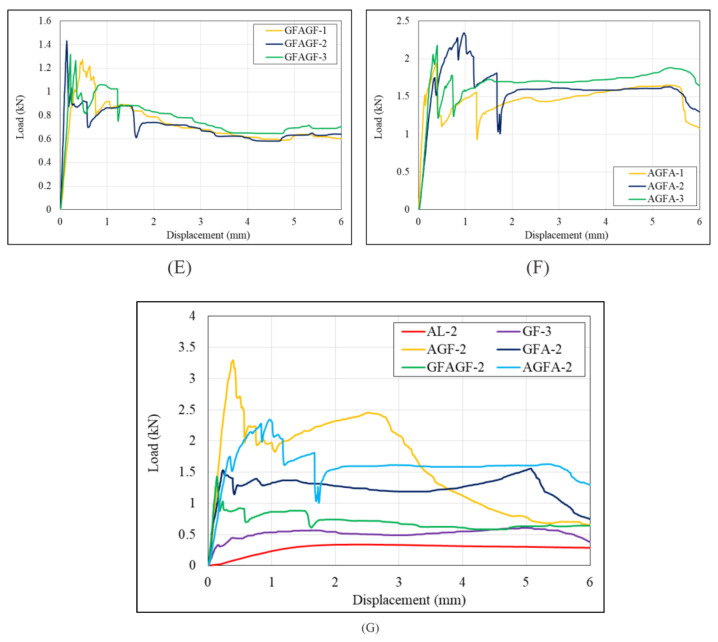
Load and displacement curve of (**A**) aluminum, (**B**) GFRP, (**C**) AGF, (**D**) GFA, (**E**) GFAGF, (**F**) AGFA, (**G**) Combined.

The lateral compression tests of all specimens are illustrated in Figure 11 and Figure 12. The data showed that, in all parameters, AGF reached the highest value: it had a PCF of 2.98 kN, a load average of 1.65 kN, energy adsorption of 10.41 J, and SEA of 0.89 Jg^−1^. Only one parameter showed a different pattern in CFE, where the highest value occurred at GF with 1.01.

Similar patterns could be observed between each hybrid stacking sequence variation in the lateral compression load-displacement curves. The load rose linearly until it reached peak force, whereafter it fluctuated wildly, forming multiple peaks and bottoms, stabilized, and went downwards toward the end of the test. However, no significant difference in the load-displacement curve could be observed between the net aluminium and net GFRP specimens. Both net specimens’ loads decreased gradually after reaching the initial peak crushing force toward the end of the test. One of the differences that can be observed between the two is that the net GFRP specimens had a slight rebound of around 4 mm of displacement, forming the second peak. Another difference is that the net aluminium curve was smoother, with less serration than the net GFRP curve.

Contrary to the axial test, a considerable difference in load carrying and energy absorption capacity could be observed between the net GFRP specimens and the hybrid specimens. The hybrid specimens surpassed the net GFRP in almost every crashworthiness indicator, starting from the initial peak force, average load, energy absorption, and specific energy absorption. The net GFRP had a higher CFE value due to having a second peak that was higher than the initial peak, as mentioned earlier.

When comparing the hybrid specimens, some differences could be observed from the load-displacement curve of each stacking sequence. The GFA and AGFA specimens retained the peak force better than the other two variations, where, after reaching peak force, the force stabilized without dipping sharply, like the AGF and GFAGF specimens. The crash force efficiency (CFE) could also determine peak force-retaining capabilities. Higher CFE means the average force (F_avg_) is closer to the peak force (PCF), which also means better peak force-retaining capability. This indicates that the GFA and AGFA stacking sequence could absorb energy more stably and constantly, which is a positive sign. When comparing the average value of each crashworthiness indicator, the AGF specimen had the highest value of all indicators except CFE. Despite only having one piece of aluminium component, the AGF specimen is better at load carrying, total energy absorption, and specific energy absorption than any other variation, including the AGFA. The crashworthiness indicators could explain the specimens’ behavior under lateral compression loading. With the highest PCF, AGF is best at compression strength, meaning that a higher load was required to crush. The highest total energy absorption (EA) means that AGF is also best at absorbing crash energy during its deformation.

In contrast, the highest specific energy absorption (SEA) means that the AGF sequence is the most weight-effective. Lower CFE indicates that the AGF sequence’s crushing behavior is not stable, in that the load will decrease after reaching the peak crushing force, causing wide gaps between the peak force and the average force. These indicators should be considered for selecting the most suitable stacking sequence of L-profile aluminium/GFRP hybrid composites. For vehicle crash energy-absorbing components, the AGF and AGFA stacking sequences are more favorable than the other sequences, and GFAGF is less favorable. The AGF sequence is best for absorbing the total energy of a crash, and AGFA is best for stable energy absorption throughout the crash event.

**Figure 13:**
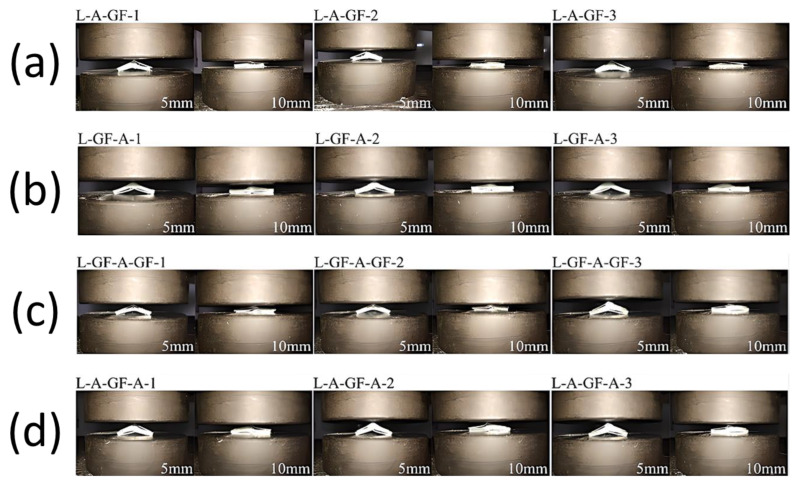
Lateral deformation history of (**a**) AGF, (**b**) GFA, (**c**) GFAGF, (**d**) AGFA.

The transverse crushing process of each specimen is shown in Figure 13. Three samples of each variation were tested, and the photos were taken at 5 mm and 10 mm crushing distances. Similar failure modes could be observed in each stacking sequence variation during the lateral compression test. Initially, the straightening deformation could be seen in every specimen. The compression load exerted by the UTM caused the specimen to bend, and the aluminium component surface started to detach from the GFRP. The specimen with aluminium on the outer side could bear a greater load but had a more catastrophic deformation once the plastic deformation started. On the contrary, the specimen with aluminium on the inner side could bear a lesser load but had a more stable deformation throughout the test. The AGFA specimen had the combined benefits of both sequences.

### 3.3. Discussion

In the axial compression test, the hybrid composites with the stacking of AGFA showed the highest energy absorption, with 157.19 J. Meanwhile, in the lateral compression test AGF had the highest energy absorption, with 10.41 J. The results corroborate the findings of Gowid et al. [26], where the best specimen in crashworthiness was a seven-layer specimen with the addition of steel. It showed the best result in initial peak force (IPF), energy (E), specific energy (SE), and crush force efficiency (CFE). The study by S. Li et al. [25] shows the influence of metal on a structure. Aluminium has a high contribution to total energy absorption. The stiffness of aluminium in the AGFA, AGF, and GFA specimens greatly benefited the axial and lateral compression tests. In this study, the specimen that had reinforced aluminium showed a better result in every aspect.

Filled structures with foam or metal have better compression behavior, in particular a more stable ability to collapse. A study done by Sun et al. [22] on Al and GF specimens without stacking had the worst number in all categories. This means that it is crucial to have a stacking sequence in terms of the compression test, axially and laterally. In the research conducted by Sebaey T et al. [27], an investigation was performed of the influence of thermal aging on the compression properties of CFRP composite rectangular tubes under a quasi-static lateral compression test, where polyurethane was the reinforcement of the rectangular tubes, resulting in a gradual increase in each of its specimen. The percentage of CFE from the aging process was increasing.

Due to the diffusion mechanism, aging causes the foam cell to lengthen and the cell pressure to rise. Following a 14-day exposure to 100 °C, the specimens’ cells began to exhibit plastic deformation due to thermal loading and the bursting of specific cells, which yielded the specimens’ strength and energy absorption. Compared to empty structures, foam-filled CFRP tubes demonstrated 60% and 25% increases in peak and average crushing loads, respectively. The same thing happened in this study, where the Al and GF specimen scored the lowest overall. A stacking sequence for the compression test is crucial, both axially and laterally. The specimen will be substantially more capable of increasing its crashworthiness by adding and changing stacking as reinforcement. A stacking sequence can increase the strength of the structure significantly. Zuo et al. [28] analyzed the crashworthiness and failure process of CFRP tubes exposed to hydrothermal aging and high temperatures. The crashworthiness features rapidly declined as temperature and moisture absorption rates rose. Hydrothermal aging was expected to improve the quality of the specimen so that the crashworthiness value increased, but the results showed otherwise. The temperature and crashworthiness indexes showed some nonlinear relationships. Compared with this study, we can increase the crashworthiness index by strengthening the structure of GF and Al. The best stacking sequence in terms of weight, efficiency, and properties is that of AGF due to its great load-carrying capacity, energy absorption, and specific energy absorption in axial and lateral loading.

## 4. Conclusions

This study presents an experimental investigation of the effect of stacking sequences on the crashworthiness of the aluminium/GFRP hybrid composite L-profile structure. The axial and lateral quasi-static compression tests investigated load-carrying capacity, energy absorption, crash force efficiency, specific energy, and crushing behavior. Net aluminium, net GFRP, and four stacking sequences of the aluminium/GFRP hybrid composite were manufactured, tested, and evaluated for crashworthiness and then compared to determine the most suitable stacking sequence for automotive energy-absorbing structure. The following conclusions can be drawn:

- In the axial compression test, the aluminium/GFRP hybrid tends to crush in a more progressive and stable failure mode compared with the net aluminium and net GFRP specimens, with a relatively more stable load-carrying capacity throughout the experimental tests. The addition of a thin aluminium structure is also effective in increasing the initial peak crushing force of the specimen, as it could be observed that every hybrid specimen had a higher PCF value than the net GFRP specimens.
- Comparing the aluminium/GFRP hybrid specimens in axial loading, it can be observed that the AGFA stacking sequence’s PCF is significantly higher than any of the other stacking sequences and its energy absorption capacity slightly higher, attributable to its having two pieces of thin aluminium structure. However, the AGF stacking sequence was second at energy absorption capacity, with a very slight difference compared to AGFA (145.31 kJ versus 157.19 kJ, respectively).
- In the lateral compression test, a significant increase in load carrying and energy absorption capacity could be observed from the aluminium/GFRP hybrid specimens over the net GFRP specimens. This indicates that adding the thin aluminium structure on the L-profile GFRP effectively increases its lateral crushing capability.
- Comparing the hybrid specimen, it could be observed that the AGF stacking sequence had the highest PCF, EA, and SEA value compared to the other specimens, despite only having one piece of thin aluminium structure. However, a few trade-offs in adding the thin aluminium structure could be observed from the other crashworthiness indicators, namely the lower specific energy absorption, caused by the extra weight of the aluminium, and the lower crush force efficiency (CFE).
- The Al and GF specimens had the worst number in all categories. It is essential to have a stacking sequence in terms of compression test, axially and laterally. By adding and varying stacking as reinforcement, the specimen’s ability for its crashworthiness will increase significantly.

The overall most crashworthy stacking sequence among the four variations tested in this experimental research was the AGF stacking sequence, due to its significant load-carrying capacity, energy absorption, and specific energy absorption in axial and lateral loading. Besides the quasi-static crushing experimental test, the dynamic performances of aluminium/GFRP hybrid tubes under impact [29] and blast loading [30,31] should also be comprehensively investigated to support this claim. The study presented in this work shows potential opportunities for developing hybrid composites with enhanced properties.

## Figures and Tables

**Table 1:** Axial compression specimen dimensions and volume fraction.

Specimen	No.	L, mm	Y, mm	W, mm	T, mm	M, g	Vf
Al	1	49.9	29.5	19	0.8	3.5	-
	2	50.1	29.5	19	0.8	3.5	-
	3	49.9	29.5	19	0.8	3.5	-
GF	1	50.3	30.1	21.65	2.6	10	0.53
	2	50.5	29.8	21.35	2.85	10	0.48
	3	50.4	30.2	21.85	2.75	10.3	0.50
AGF	1	50.4	29.6	19.3	3.25	11.4	0.56
	2	50.3	29.3	19.1	3.25	11.1	0.57
	3	50	29.4	19.35	3.3	11.4	0.55
GFA	1	50.8	29.1	22.05	3.55	13.4	0.48
	2	50.5	30.3	21.8	3.35	14	0.52
	3	49.9	29.9	21.2	3.4	12.8	0.52
GFAGF	1	50	29.5	20.7	3.5	13	0.50
	2	50.4	29.3	20.5	3.4	12.7	0.52
	3	49.9	29.6	20.1	3.6	12.2	0.49
AGFA	1	49.8	30.6	22.5	4.25	16.8	0.48
	2	50.1	30.2	22.6	4.25	17.2	0.48
	3	50	30.6	22.7	4.45	15.6	0.45

**Table 2:** Lateral compression specimen dimensions and volume fraction.

Specimen	No.	L, mm	Y, mm	W, mm	T, mm	H, mm	M, g	Vf
Al	1	50.3	29.5	18	0.8	13.7	3.5	-
	2	49.6	29.5	18	0.8	13.7	3.5	-
	3	50.1	29.5	18	0.8	13.7	3.5	-
GF	1	50.3	30.2	21.9	2.6	16.4	11.0	0.53
	2	50.4	30.7	21.9	3.0	16.1	10.9	0.46
	3	50.0	30.8	21.9	2.95	16.0	10.4	0.47
AGF	1	50.6	29.7	19.35	3.5	16.3	11.2	0.51
	2	50.3	29.6	19.05	3.4	15.9	11.5	0.53
	3	49.9	29.3	19.05	3.5	15.9	12.2	0.52
GFA	1	50.2	30.6	22.2	3.85	16.9	15	0.44
	2	50.1	30.3	21.5	3.85	16.7	14.7	0.44
	3	50.2	29.0	22.3	3.6	16.5	15	0.48
GFAGF	1	50.2	29.6	20.85	3.5	16.1	13.3	0.50
	2	50.2	29.6	20.7	3.8	16.1	13.5	0.45
	3	50.2	29.9	20.6	3.4	15.9	13.3	0.52
AGFA	1	49.9	30.6	23	4.75	19	18.1	0.41
	2	49.8	30.3	22.9	4.8	18.9	17.2	0.40
	3	49.9	30.7	22.95	5.15	19.2	17.9	0.37

## Data Availability

Data will be available upon request.

## Appendix A

### Appendix A.1. Experimental Result of Axial Compression Test

**Table A1:** Axial crashworthiness indicators of each specimen.

Specimen	No	*PCF,* kN	F_avg_, kN	EA, J	SEA, J.g^−1^	CFE
Al	1	2.43	0.76	4.59	1.31	0.31
	2	2.17	0.70	4.18	1.19	0.32
	3	2.30	0.73	4.37	1.25	0.32
GF	1	8.04	5.95	117.07	11.71	0.74
	2	8.39	7.21	143.16	14.32	0.86
	3	8.14	8.13	159.94	15.53	1.00
AGF	1	13.70	8.21	164.29	14.41	0.60
	2	12.98	5.40	108.04	9.73	0.42
	3	13.95	8.18	163.59	14.35	0.59
GFA	1	12.45	6.99	139.79	10.43	0.56
	2	10.22	7.17	143.37	10.24	0.70
	3	11.61	5.37	107.36	8.39	0.46
GFAGA	1	13.08	4.72	94.37	7.26	0.36
	2	15.85	4.46	89.22	7.02	0.28
	3	15.88	4.28	85.55	7.01	0.27
AGFA	1	25.30	8.81	176.16	10.49	0.35
	2	26.81	6.97	139.42	8.11	0.26
	3	21.67	7.80	156.00	10.00	0.36

**Table A2:** Comparison of the average axial crashworthiness indicators.

Specimen	*PCF,* kN	F_avg_, kN	EA, J	SEA, J.g^−1^	CFE
Al	2.30	0.73	4.38	1.25	0.32
GF	8.19	7.09	140.06	13.85	0.87
AGF	13.54	7.43	145.31	12.83	0.55
GFA	11.42	6.51	130.17	9.69	0.58
GFAGF	14.94	4.49	89.71	7.10	0.30
AGFA	24.59	7.86	157.19	9.53	0.32

### Appendix A.2. Experimental Result of Lateral Compression Test

**Table A3:** Lateral crashworthiness indicators of each specimen.

Specimen	No	*PCF,* kN	F_avg_, kN	EA, J	SEA, J.g^−1^	CFE
Al	1	0.33	0.24	1.62	0.46	0.75
	2	0.34	0.25	1.68	0.48	0.74
	3	0.32	0.27	1.62	0.46	0.82
GF	1	0.39	0.44	2.61	0.24	1.13
	2	0.46	0.46	2.70	0.25	0.99
	3	0.56	0.52	3.10	0.30	0.92
AGF	1	2.45	1.54	10.05	0.90	0.63
	2	3.30	1.53	9.67	0.84	0.46
	3	3.20	1.89	11.52	0.94	0.59
GFA	1	1.27	1.19	7.02	0.47	0.94
	2	1.53	1.24	7.47	0.51	0.81
	3	1.03	1.18	7.01	0.47	1.15
GFAGF	1	1.27	0.73	4.33	0.33	0.58
	2	1.43	0.71	4.23	0.31	0.49
	3	1.32	0.78	4.62	0.35	0.59
AGFA	1	1.95	1.47	8.76	0.48	0.76
	2	2.35	1.60	9.64	0.56	0.68
	3	2.18	1.70	10.06	0.56	0.78

**Table A4:** Comparison of the average lateral crashworthiness indicators.

Specimen	*PCF,* kN	F_avg_, kN	EA, J	SEA, J.g^−1^	CFE
Al	0.33	0.25	1.64	0.47	0.77
GF	0.47	0.47	2.80	0.26	1.02
AGF	2.98	1.65	10.41	0.89	0.56
GFA	1.28	1.21	7.17	0.48	0.97
GFAGF	1.34	0.74	4.40	0.33	0.55
AGFA	2.16	1.59	9.49	0.54	0.74
